# Conducting cross-sectoral collaborative research for food system transformation: learnings from a qualitative evaluation study

**DOI:** 10.1186/s12961-026-01540-y

**Published:** 2026-10-06

**Authors:** Sarah Shaw, Oliver Huse, Bea Savory, Noah Cooke, Claire Thompson, Rachel Loopstra, Peter Scarborough, David Ogilvie, Steven Cummins, Martin White, Jean Adams

**Affiliations:** 1 MRC Epidemiology Unit, School of Clinical Medicine, University of Cambridge, Cambridge, CB2 0QQ United Kingdom; 2 Population Health Innovation Lab, Department of Public Health, Environments & Society, London School of Hygiene & Tropical Medicine, 15-17 Tavistock Place, London, WC1H 9SH United Kingdom; 3 University of Hertfordshire Centre for Research in Public Health and Community Care, Hatfield, United Kingdom; 4 Department of Public Health, Policy and Systems, Institute of Population Health, Faculty of Health and Life Sciences, University of Liverpool, Liverpool, United Kingdom; 5 Nuffield Department of Primary Care Health Sciences, University of Oxford, Radcliffe Observatory Quarter, Woodstock Road, Oxford, OX2 6GG United Kingdom; 6 School of Geography and Environmental Science, Faculty of Environmental and Life Sciences, University of Southampton, SO17 1TJ Southampton, United Kingdom

**Keywords:** Food system interventions, Evaluation, Qualitative, Co-designed research

## Abstract

**Background:**

The SALIENT consortium aimed to bring together research, government and food system stakeholders to co-design, implement and evaluate public-facing food system interventions targeting health and sustainability whilst maintaining affordability and commercial viability. A total of 40 organizations engaged with SALIENT; 8 progressed to deliver and evaluate a total of 10 interventions. We conducted an evaluation to understand how and why the SALIENT stakeholder engagement process worked for different organizations and to inform future food system intervention research.

**Methods:**

Using theory-based evaluation principles, a theory of change (ToC) was developed to describe how the SALIENT process was expected to function. A total of 38 semi-structured interviews were conducted with government partners, researchers and food system representatives. Data were thematically analysed and interpreted in relation to the ToC.

**Results:**

Findings highlighted the fragmented nature of existing health and sustainability actions across the food system, shaped by differing priorities, agendas and constraints of stakeholders. Power dynamics influenced stakeholder participation, with engagement strongest when research was aligned with core stakeholder goals and offered them tangible benefits. Whilst pragmatic co-design processes built trust and feasibility, they sometimes led to interventions with limited potential to address critical uncertainties in the evidence base. Academic processes were often perceived as misaligned with stakeholders’ practical needs.

**Conclusions:**

Long-term government direction, stronger accountability, and incentives are needed to foster meaningful collaboration between researchers and food system stakeholders. Future partnerships should balance flexibility with rigour, build trust through sustained relationships, and establish strong governance, communication and evaluation processes early to generate robust, scalable evidence for meaningful food system change.

**Supplementary Information:**

The online version contains supplementary material available at https://doi.org/10.1186/s12961-026-01540-y.

## Background

The food system in many high-income countries, including the United Kingdom, is detrimental to both human and planetary health [1]. Widespread availability and heavy marketing of foods high in salt, fat and sugar are a major driver of noncommunicable diseases, disproportionately affecting those from the most disadvantaged backgrounds [2]. Food insecurity is also common, with one in six households struggling to afford sufficient food every month [3]. At the same time, current food production methods contribute significantly to water pollution, biodiversity loss and greenhouse gas emissions [1, 4, 5].

Re-orientating the food system to optimize benefits for both people and the planet remains a complex and intractable problem that needs to be underpinned by robust evidence. To meet these evidence needs, there have been calls for the formation of new research collaborations that connect multiple scientific disciplines and engage in co-production with a diverse range of food system stakeholders [6, 7]. In co-produced research, researchers work in close partnership with stakeholders throughout the research process to ensure work is relevant, impactful and grounded in multiple forms of knowledge [8–10]. Co-production approaches emphasize shared learning, mutual respect and equitable power, generating research that better addresses real-world needs in a way that is amenable and acceptable to all contributors. Such approaches are believed to improve the likelihood of research findings becoming embedded in policy and practice [10]. Engaging multiple stakeholders in research, however, is not without its challenges. Previous studies highlight barriers relating to geographic dispersion, conflicting priorities and timescales, power imbalances, limited face-to-face interaction and varying levels of commitment, as well as system-level challenges such as the restrictive nature of research funding and academic systems that are not aligned with “real-world” practice [11–13].

The practicalities of co-production within the realities of existing food systems remains largely underexplored. The food system is a complex, adaptive network made up of interconnected subsystems and a diverse range of stakeholders, each with differing agendas, priorities and levels of influence [14]. The food system is dominated by commercial actors, whose priorities largely shape food production, distribution and retail practices. In contrast, the community food sector, referring to third-sector organizations who work outside the commercial food system to provide food at no or low cost to people experiencing food insecurity, is relatively small, used by an estimated 7% of households in the United Kingdom [15]. Government actors shape the system through policymaking, regulation, funding and priority-setting across government departments addressing health, welfare, agriculture, employment, economy and the environment. Researchers, typically from universities, contribute by generating knowledge and evidence to inform and improve actions within the food system. The purposes, values and interests of food sector, government and academic stakeholder groups are not always aligned with one another, making it difficult to work towards a common objective [16]. In particular, many commercial food organizations rely on the sale of foods made with unhealthy and unsustainable ingredients to generate profit, whereas achieving public health and sustainability goals may require reduced manufacture, purchasing and consumption of these products [16, 17]. More recently, a smaller number of commercial organizations with impact-oriented business models, some aligned with health and sustainability targets, have emerged, although such approaches are yet to become the norm [18].

The SALIENT Consortium was established in response to an Economic and Social Research Council funding opportunity for academic researchers to work closely with government and commercial and community food system stakeholders to generate robust evidence regarding the effectiveness of interventions to encourage and enable affordable, healthier and more sustainable diets [19]. The Consortium was initially awarded 6 months of co-design funding followed by a 22-month delivery grant. A non-costed extension was also received to extend the project by a further 12 months. During this time, members of the Consortium engaged representatives of more than 40 commercial and community food organizations through online and in-person meetings and workshops with the aim of developing a range of co-designed interventions and/or evaluations. During the co-design period, some organizations decided not to work with the SALIENT Consortium (referred to as non-participating stakeholders). Due to time and resource limitations, and as anticipated from the outset of the SALIENT project, all potential intervention evaluation opportunities could not be carried forward and included in the final set of SALIENT studies. A prioritization process was adopted whereby the research team members voted on intervention and evaluations to carry forward based on criteria relating to engagement, equity implications, transformative potential and risks. In total, 10 interventions have been evaluated in collaboration with eight commercial and community food system partners (see: https://www.studies.epi.ims.cam.ac.uk/salientfoodtrials/about.

Alongside intervention-specific evaluations, the SALIENT programme provides a unique opportunity to draw learning from the process of the work itself. We aimed to conduct a qualitative evaluation of the SALIENT research programme to understand how effectively government, academic and food system stakeholders collaborated to co-design, implement and evaluate a range of public-facing food system interventions focusing on health, sustainability and affordability. Specifically, we aimed to address two research questions:

1. How and why did the SALIENT stakeholder engagement process work for some SALIENT partner organizations and not for others?
2. What can we learn from the SALIENT stakeholder engagement process to improve similar future efforts?

## Methods

### Study design

**Fig. 1:**
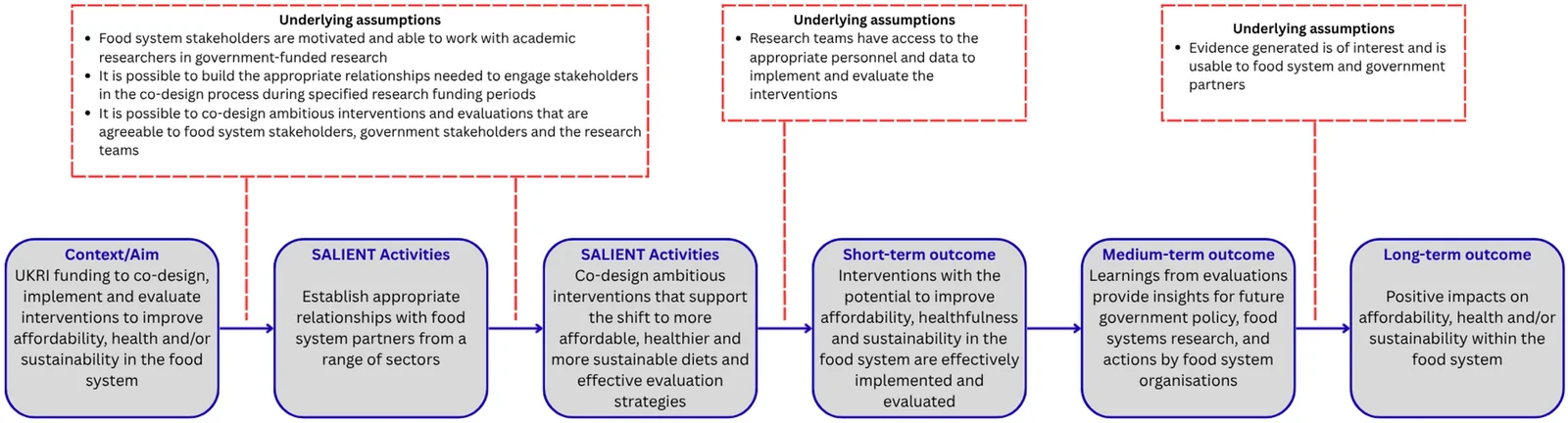
Condensed theory of change showing logic chain and underlying assumptions on how collaborative food systems research functions and may lead to benefits

We used a qualitative study design to address the research questions. Using theory-based evaluation principles, such as contribution analysis [20], a theory of change (ToC) was developed to describe how the work of the SALIENT research programme was hypothesized to generate evidence about interventions aiming to improve affordability, health and sustainability within the food system (Additional File 1). This ToC was developed by S.S. (the lead author) and refined through input first from co-authors, and then from the entire SALIENT Consortium. The ToC consists of a logic chain representing how SALIENT activities were connected and could result in multiple outcomes over the short and long term, and identifies underlying assumptions required for it to hold. This ToC was used to develop and structure topic guides for the stakeholder interviews. The ToC was subsequently condensed to enable relevant insights for other food systems research to be generated and used to interpret the qualitative findings of this study. Figure 1 shows the condensed ToC. In reporting this study, we followed the Consolidated Criteria for Reporting Qualitative Research (COREQ) [21].

### Participants and recruitment

We conducted primary qualitative interviews and incorporated further data from interviews conducted during SALIENT intervention process evaluations. Four stakeholder groups were represented in the data. Primary data were collected from:

1. Programme board members: representatives from government departments and the funding body who served on the Food Systems Trials Programme Board overseeing the research programme. Purposive recruitment focused on members most actively involved and representing a range of government departments.
2. Consortium members: members of the SALIENT research team who were responsible for developing, implementing and evaluating the interventions. Purposive recruitment focused on different career stages and research roles in the consortium.
3. Non-participating food system stakeholders: representatives from food system organizations that engaged but did not progress to evaluating an intervention with SALIENT. Decision-makers from these organizations, such as senior executives and managers, were invited to participate and purposive recruitment aimed to include different food sectors and levels of engagement. Decision-makers were included as these were the individuals most involved in the conversations with the research team about participation and co-design.
  Secondary data from SALIENT process evaluation interviews were drawn from:
4. Participating food system stakeholders: decision-makers from food system organizations who engaged with SALIENT to implement and evaluate an intervention.

For the primary evaluation interviews, potential participants were identified from SALIENT records. Invitations were sent via email either by S.S., or for food system stakeholders, by the SALIENT Consortium member who had most recently been in contact with them. Those who wished to participate were asked to reply to the invitation email.

We also included data from interviews conducted with decision-makers from participating organizations. During the process evaluation interviews, these individuals were also asked additional questions specific to this evaluation study. Data from the full interviews were included in the current analysis.

In total, data from 38 interviews with 40 participants were included: 30 primary evaluation interviews and 8 process evaluation interviews. Interviews were conducted one-on-one, except for one process evaluation group interview, which involved three participants from the same organization. Details of the study participants can be found in Table 1.

**Table 1:** Overview of interview participants

Stakeholder group	*n*^a^		Sector	*n*^b^	Participants’ organizational roles	*n*^1^
Programme board		5	Government department representatives	4	Research-related position Food/nutrition position	3 2
			Funding body representatives	1		
Research team		16	University	14	Senior researchers Research staff*	9 5
			Third-sector research organization	2	Co-applicants* Research staff*	1 1
Non-participating food system stakeholders		9	Commercial stakeholders	6	Executive position	1
			Out of home sector	2	Nutrition and/or sustainability role	5
			Retailers	3		
			Catering	1		
			Community stakeholders	3	Executive position	1
			Food aid organization	2	Research/advisory position	2
			Food advocacy organization	1		
Participating food system stakeholders		10	Commercial stakeholders	4	Executive position	1
			Retailers	2	Nutrition and/or sustainability role	1
			Technology firm	2	Impact director	1
					Head of product	1
			Community stakeholders^*^	4	Executive position	1
			Food aid organization	3	Head of impact	1
			Social supermarket	1	Site manager	2
					Research role	2

^a^*n* in this column represents number of participants

^b^*n* in this column represents number of interviews

*Research team co-applicants were named on the funding application and contributed to both securing funding and designing the project. Research staff were employed to deliver the project but were not named on the funding application and were generally more junior researchers

### Data collection

Interviews were conducted on Microsoft Teams by S.S. Process evaluation interviews were conducted on Zoom or in person by O.H. or B.S.

A semi-structured topic guide (Additional File 2) was created for this study. The ToC informed the development of the semi-structured topic guide. Interview questions were mapped to key elements of the ToC, including the activities undertaken within SALIENT, the mechanisms through which collaborative food systems research was expected to influence outcomes and stakeholder experiences of engagement and implementation. The topic guide was developed by S.S. and refined through discussion with co-authors to ensure relevance across the different stakeholder groups included in the evaluation. The process evaluation interviews incorporated questions from this topic guide to ensure the same topics were covered. Interviews lasted 30–60 min and were conducted between April 2024 and January 2025. This timepoint captured varying stages of the SALIENT process: some interventions were still under negotiation, whilst others were already being evaluated.

### Analysis

Interviews were transcribed verbatim by a trusted transcription agency and later checked by the research team. Transcripts were anonymized to remove references to individuals and participating organizations.

The analysis was led by S.S., with critical feedback provided by co-authors throughout the process. The analysis followed the six-phase process of reflexive thematic analysis outlined by Braun and Clarke [22, 23]. After initial data familiarization, S.S. used NVivo software to inductively code interview transcripts. This initial coding process commenced whilst interviews were still ongoing. Given the iterative nature of reflexive thematic analysis, S.S. repeatedly moved between the phases, revisiting earlier stages as new insights were identified. Following initial coding, S.S., J.A., D.O., S.C. and M.W. met to critically reflect on the developing analysis, discuss identified patterns and ensure that the themes were both clearly defined and grounded in the data. Based on this collaborative discussion, S.S. further refined the analysis to ensure the themes were clearly defined and grounded in the data. This process led to the development of three overarching themes, which were subsequently interpreted through the lens of the condensed food system research ToC (Fig. 1). All authors provided feedback on the final themes and data interpretation.

### Reflexivity statement

Work conducted by the SALIENT consortium is grounded in the belief that it is beneficial to improve the food system by providing increased access to foods that are supportive of health, have lower environmental impact and are affordable to all. The research team also recognizes that, for the commercial sector to voluntarily introduce interventions, those interventions must be commercially viable to ensure adoption and sustained implementation. This evaluation was conceived and conducted by an interdisciplinary team of academic researchers, many of whom have prior experience working with food system stakeholders in health-related research. The researchers share a commitment to population-level interventions aimed at advancing equitable access to healthy and environmentally sustainable food, and they view government policy as a critical lever in achieving this goal. These experiences have informed their understanding of both the value and complexity of stakeholder engagement in such initiatives. These perspectives may have influenced interpretations of stakeholder engagement and motivations, particularly where commercial priorities and public health objectives differed. During analysis, the team therefore sought to remain attentive to participants’ accounts and avoid imposing predetermined assumptions about the roles or motivations of different sectors.

The lead author for this work (S.S.) joined the project partway through the SALIENT funding period, whilst the senior author (J.A.) was a co-applicant on the SALIENT grant. S.S. was deliberately recruited at this stage to provide a more independent perspective on how the consortium functioned. However, S.S. was also embedded within the wider research team and therefore was not a complete outsider. Throughout analysis, S.S. discussed emerging interpretations with co-authors to critically examine assumptions and alternative explanations.

As the study aimed to include the perspectives of all SALIENT stakeholders, some authors of this paper were also study participants. This insider position provided valuable contextual understanding of the research process but also introduced the possibility that personal experiences could influence interpretation of the findings. To mitigate this, participant-authors were not responsible for interpreting their own accounts, and analysis involved discussion amongst authors with different levels of involvement in SALIENT. Reflexive discussions focused on ensuring that no individual perspective dominated the interpretation and that themes remained grounded in the wider dataset.

## Results

Three themes were identified from the data and are outlined in Table 2 together with subthemes. Descriptions of the themes are provided with illustrative quotes. Some quotes have been slightly edited to remove superfluous words without changing the overall meaning.

**Table 2:** Overview of themes and subthemes

Food system transformation requires clear vision and a coordinated long-term strategy
1. Large variations in current actions and goals for health and sustainability across the food sector
2. Food system stakeholders vary in their level of enthusiasm and commitment for further actions on health and sustainability
3. Different agendas across government departments lead to varying levels of commitment to food system transformation
4. Many ambitions for SALIENT were challenging to achieve in practice, but progress has been made
Power structures and organizational priorities shape stakeholder engagement in food systems research
1. SALIENT provided an opportunity to deliver on secondary organizational targets so long as these did not clash with core organizational goals
2. Decision-making power is unlikely to lie within health and sustainability teams in commercial organizations
3. Government power needs to be appropriately harnessed to encourage stakeholder action
4. Power dynamics amongst stakeholders when co-designing interventions can limit ability to address critical uncertainties in the evidence base
Preferred ways of conducting research varied according to stakeholder type
1. Food system stakeholders varied in their perceptions of the usefulness of academic research for their organization
2. The perceived risks associated with academic collaboration differ depending on food sector
3. The pace of academic research does not align with the pace of activities carried out by food system organizations

### Theme 1: Food system transformation requires clear vision and a coordinated long-term strategy

Stakeholders described food system transformation as a complex and fragmented process, with substantial variation in organizational priorities, ambitions and capacity to engage in health and sustainability activities. This theme describes how differing objectives across food system actors, alongside changing policy contexts and the limited timeframe of SALIENT, influenced the extent to which collaborative action could be achieved.

#### Subtheme 1.1: Large variations in current actions and goals for health and sustainability across the food sector

Most food system stakeholders described some existing health and sustainability actions within their organizations, but these were approached with different levels of priority, ambition and commitment, with many describing these actions in ways suggesting that they were peripheral to the core functioning and goals of their organizations.

Every single individual retailer will have their own vision of what they think that food transformation is, and that’s the missing bit, because actually, whilst we can have our vision, visions across retail will be very, very different (non-participating (NP) commercial stakeholder 3).

The nature of the actions being undertaken varied substantially across organizations. Some commercial organizations described actions reflecting recent government policy whilst others described a diverse range of activities relating to health or sustainability. Some strategies and targets were publicly available whilst others were internal, limiting the potential for cross-organizational alignment.

That can be anything as broad as where we’re working with our farmers to improve the emissions coming out of our beef, to anything to do with increasing the use of wholegrains or fruit and veg, and going back to the Eatwell plate in our customer comms or development to make sure that we offset some of our protein. So, I suppose it’s anything from a communications tool, to going back to basics, to a product development tool, to actually working with our suppliers (NP commercial stakeholder 3).

#### Subtheme 1.2: Food system stakeholders vary in their level of enthusiasm and commitment for further actions on health and sustainability

Stakeholders’ willingness to engage with SALIENT was shaped by their organizational priorities, commercial pressures and capacity for additional work. Commercial representatives who had invested heavily in complying with recent government regulations around healthy food showed limited interest in further immediate action in this area due to fears of undermining their competitiveness and provoking consumer backlash. Whilst some stakeholders initially showed interest in engaging with SALIENT, this often faded due to competing demands, with limited commitment to follow through on proposed ideas.

I think the expectation is that the industry has unlimited resource, when at the end of the day we don’t, the food industry is really competitive, it’s a weird market the UK. But we don’t have massive margins, and we don’t have tons of money to spend on this stuff, so we spend a lot of time implementing HFSS [high fat, sugar and salt] regs, most of my days is implementing HFSS regs, or EPR [extended producer responsibility] regs, or whatever it is we’re working on next (NP commercial stakeholder 5).

Members of the research team also noted a perceived preference amongst commercial stakeholders to engage in sustainability, rather than health issues. They described more positive interactions in this area, as goals related to sustainability were seen as more established and attainable.

Oh, I’d say, broadly, sustainability is kind of viewed as more of a priority, they have a pressure on that, they have reporting guidelines that they need to make… I think it’s mostly the sort of corporate social pressure stuff that is more developed on sustainability type goals (Consortium member 9).

Some community organizations viewed health and sustainability as integral to tackling poverty and food insecurity. Others, particularly those not participating in SALIENT, saw these issues as drivers of demand for their services rather than outcomes that they could meaningfully influence. For many, the priority was to uphold dignity and eliminate the need for food aid, rather than focus on improving the nutritional quality or sustainability of the food provided.

[...] the primary thing from our perspective, this really seemed to be a project really about health and improving people’s diets, and also in a way it was about improving the content of what’s delivered through food aid, and our focus is on, not on really improving food aid, it’s on how do we end the need for food aid, and so there was a risk of like conflicting messaging for us (NP community stakeholder 2).

Community stakeholders who did participate shared the view that reducing the demand for food aid was the ideal long-term solution but saw it as beneficial within the current system to improve the healthfulness of the food they provide.

I’d like to think as a part of [our work], that we provide good, healthy food for people that can sustain their families, and that was a large part of the motivation for doing the work (participating community stakeholder 1).

#### Subtheme 1.3: Different agendas across government departments lead to varying levels of commitment to food system transformation

Programme board members came from multiple government departments and had varying priorities. Some departments who were originally part of the programme board become disengaged when the research was seen as not directly relevant to their policy interests. In other cases, some members who were more engaged expressed frustration that SALIENT did not generate evidence aligned with their interests.

So there was a bit of conversation around tensions between different government partners in terms of what SALIENT was delivering; I think there was a bit of, discontent in that respect, and some government partners have been more involved than others, so for instance [government department] is now basically a silent partner because none of the food trials are focusing on [policy area] (Programme Board (PB) member 4).

Some programme board members recognized that government priority areas changed with time, and this was particularly the case with the change of government that occurred during the funding period. This made it difficult for SALIENT research to meet the changing areas of policy interest.

I think one of the challenges as well, it’s worth bearing in mind, is the Government departments’ priorities change over time. You know, you’ve got a new Government at the moment, but we’ve had some ministerial reshuffles and things like that, so there’s a changing set of priorities (PB member 3).

#### Subtheme 1.4: Many ambitions for SALIENT were challenging to achieve in practice, but progress has been made

Some participants acknowledged that the high level of ambition expected by some stakeholders was not possible to deliver within the current food system and programme constraints. Members of the research team and programme board believed the original funding call was primarily oriented towards working with large partners to test high-impact interventions at scale, but the challenges of doing so were not fully recognized at the outset by all stakeholders. Whilst some participants expressed disappointment and frustration that such large-scale partnerships were not achieved, others were less surprised and saw this as an inevitable outcome of the research.

I think, the mindset of Government we went in with, “Great, we can do something transformational to the food system with the big players”, you know, and obviously we haven’t, but I think that’s where we came from, and maybe we were naïve…and then [research team member] kind of went, “Well, they’ve [industry partners] got no incentive whatsoever without legislation”. It’s like, okay, well, we didn’t know that (PB member 1).

Stakeholders recognized that SALIENT was generating valuable findings that would contribute to the larger evidence base needed to inform transformational change. Some emphasized the value of insights gained regarding the level of time and resources required to effectively engage with food system stakeholders.

A lot of work has gone into those partnership and designing those [interventions] and I wonder, but that’s for the academic group to answer, whether the investment required in designing and creating those partnerships was much more than they actually realised that it would be, but that’s an important learning [for government] in itself is how much, how much time it takes to build up that trust and develop a workable proposal (PB member 2).

Others framed the project as valuable because it enabled the testing of interventions that might not otherwise have been funded, generating important proof-of-principle evidence. Overall, most stakeholders viewed the evidence generated by SALIENT as contributing to a larger literature that could eventually inform longer-term change, particularly if successful interventions were scaled, rather than definitive in themselves.

That, there’s a kind of a tension there in terms of what the funder pushed us to do didn’t quite match with what the rhetoric in their original call and they asked for things to transform the food system but we were probably never going to be able to evaluate those kinds of interventions within that short timescale (Consortium member 12).

I don’t think SALIENT itself is going to produce anything that’s itself transformative, but I do feel like if we demonstrate value through case studies and then we can recommend, okay let’s explore what that might look like as a slightly bigger, a scaled-up version, then it has potential, but it won’t be done within SALIENT (Consortium member 15).

### Theme 2: Power structures and organizational priorities shape stakeholder engagement in food systems research

Stakeholder engagement was shaped not only by willingness to participate, but also by organizational incentives, decision-making structures and existing power relationships within the food system. This theme explores how these factors influenced who engaged with SALIENT, which interventions were considered feasible, and the extent to which collaborative processes could address wider evidence gaps.

#### Subtheme 2.1: SALIENT provided an opportunity to deliver on secondary organizational targets so long as these did not clash with core organizational goals

Many participating stakeholders described SALIENT as providing an opportunity to advance secondary organizational targets (for example, corporate social responsibility or policy engagement), many of which were framed around health and/or sustainability. However, they were only able to pursue these through actions that did not conflict with core organizational priorities (for example, maximizing sales, profit, customer satisfaction or the delivery of food aid). Participation in SALIENT frequently depended on the strength of the organization’s existing commitment to these secondary targets.

And of course, health and environment are officially goals of these companies, [retail company] has specific sustainability targets, but of course profits are too. They [health and sustainability goals] are very much secondary…because there are going to be instances where they conflict, and if it is true, which certainly is the impression they gave, they’re never ever willing to compromise even small amounts of profit, to achieve sustainability and health goals (Consortium member 2).

Stakeholders who expressed the strongest interest in participating in SALIENT often described clear and well-defined commitments to social goals. For example, the opportunity to contribute towards B-Corp accreditation was considered a motivating factor. Without tangible benefits or commitment to well-defined targets aligned with SALIENT interventions, organizations were unlikely to engage.

Being able to say that you worked with a research team gets you B Corp points. So, we’d already had it put into our minds that this would be a good move (NP commercial stakeholder 2).

#### Subtheme 2.2 Decision-making power is unlikely to lie within health and sustainability teams in commercial organizations

Research team members identified many of their primary organizational contacts as individuals from health, nutrition and sustainability teams, especially in large commercial organizations. Initial discussions with health and sustainability teams were mostly viewed as positive but often broke down once commercial or sales teams became involved, frustrating both researchers and health and sustainability contacts.

We’d started to shortlist, 10, 15 ideas for interventions. We were all very excited. So, we set a meeting with the trading team, our Commercial Director and Head of Analysis…When we got into the meeting, it became very apparent very quickly that sustainability had not given [the] commercial [team] enough heads up (NP commercial stakeholder 2).

Having the commitment and buy-in from the executive level of organizations aided SALIENT relationship-building, as their endorsement helped reassure other staff, reducing perceived risks associated with engaging in the project. Accessing these executives was easier in smaller companies as there were fewer organizational layers and more straightforward company structures. As such, support from the top for health and sustainability-promoting interventions could trickle down in smaller organizations and have a positive impact at planning and implementation stages. Whilst researchers may have perceived these smaller organizations as easier to work with, they also recognized that some offered limited potential for future rapid scale-up, as findings may not be generalizable to other organizations.

You know, with [smaller retail partner], we could get access to the CEO, the person we’re talking to just goes and has a meeting with them immediately, they can get sign-off really quickly and they can just be much more agile, these smaller companies. Whereas with [larger retail partner], I don’t know how many levels we were before we got to the kind of decision-making, but we were a long way off the decision-making body (Consortium member 16).

#### Subtheme 2.3 Government power needs to be appropriately harnessed to encourage stakeholder action

The involvement of government partners in SALIENT was initially expected to increase interest in participation. However, the research team felt that support and buy-in was needed from a much higher level of government to be effective in encouraging participation, especially for commercial food system stakeholders. In particular, the research team and programme board felt that the largest and most influential food system stakeholders would only be interested if the message to participate came from the highest, political parts of government.

Well, we wondered whether Government could twist their arms and they did try and do that, they wrote to various people but to be honest, if a CEO of a blue chip company gets a letter from a civil servant they’re not going to do anything, they only really talk to like the Chancellor of the Exchequer or the Prime Minister, you know, someone really senior in Government. So, I didn’t ever hold out much hope for that to be honest (Consortium member 12).

The programme board members and research team were clear that SALIENT should be focused on enticing voluntary participation of food system stakeholders and a hope that stakeholders would want to be involved and have their own research questions to test with researchers. However, there were mixed perspectives about how realistic this was, particularly in relation to commercial partners.

You could say if the retailers were mandated to work with SALIENT what would have happened, yeah of course it would be different, they would have engaged in something, whether you could still do something seismic is, is a different question, but yeah, it would have just changed the nature of the work. This was about partnership and this was about willingness… so that [lack of willingness] is a really important finding, isn’t it? (PB member 2).

#### Subtheme 2.4: Power dynamics amongst stakeholders when co-designing interventions can limit ability to address critical uncertainties in the evidence base

Commercial stakeholders were often described as holding significant power in discussions about potential interventions and their involvement in SALIENT. To secure participation, research teams frequently adopted pragmatic and flexible approaches to co-design. Some team members reflected on the tension between gaining access to large-scale partners and the compromises this required, often limiting the scope of interventions to those considered feasible or acceptable by commercial partners. Whilst food system stakeholders appreciated the pragmatic co-design process and felt their views were valued, this pragmatism sometimes led to intervention components being scaled back or excluded altogether. As a result, more ambitious or novel options were often set aside, which limited the ability of the research team to gather evidence to address critical uncertainties in the existing evidence base.

We did spend some time in discussion about whether it’s better to just say no and record that as an industry partner who is not prepared to evaluate a potentially effective intervention or should we go with that industry partner and generate evidence on the likely ineffectiveness of the intervention? So it’s a difficult one (Consortium member 8).

Some community organizations highlighted negative power imbalances in past partnerships with academic teams, where they felt their concerns and needs had not been adequately considered. In contrast, they viewed SALIENT’s pragmatic approach to finalizing interventions positively, though questions of equipoise arose as some community stakeholders were clear about the type of interventions they were willing to implement as they wanted to ensure benefit for their service users.

[Researcher] has been really good to work with. I think the basis by which [they] formulated those interventions, it’s been co-formulated with ourselves. [They’ve] done lots of work, not just through literature. He’s actually took the time with his team to speak to the members, speak to the actual team about what can work, what can’t (participating community stakeholder 1).

Working with intermediates, such as technology companies, to deliver interventions without the involvement of large retailers, was considered a novel and promising area for future work, and a method of circumventing the power these organizations hold.

But having looked at the trials that go forward, and ones that haven’t gone forward, it seems like it’s almost easier to develop trials through an intermediary, as opposed to directly with organisations, just became either timelines, or ways of working, or reputational issues of having an academic paper published on [major supermarkets]’s done this, and it doesn’t do anything (Consortium member 13).

Existing relationships were seen as highly beneficial when initiating discussions about SALIENT and developing intervention ideas. Established trust helped minimize perceived power imbalances and enabled researchers to more openly advocate for interventions they considered to have the greatest potential for positive change.

### Theme 3: Preferred ways of conducting research vary according to stakeholder type

Stakeholders differed in their expectations of what research should achieve, how quickly it should operate, and the forms of evidence considered most useful. This theme examines tensions between academic approaches to generating robust evidence and stakeholder preferences for timely, practical and context-specific knowledge.

#### Subtheme 3.1: Food system stakeholders varied in their perceptions of the usefulness of academic research for their organization

Many food system stakeholders acknowledged the rigour of academic research, though some felt its depth was not always necessary for their needs. Community organizations and smaller businesses valued academic expertise most, as they lacked these skills in-house. In contrast, larger commercial organizations, with their own research teams, saw less need for external input. For some stakeholders, however, the opportunity to be associated with a well-known university was motivating in itself, providing a form of validation for their work.

Various stakeholders acknowledged that academic research does not always align with practical needs. Some felt its insights offered limited value to their organizations, describing academic approaches as rigid and ill-suited to the rapidly changing priorities they faced. Some suggested this disconnect may be reinforced by funding constraints, which restrict the ways research resources can be used to deliver what they see as most beneficial to their work.

Even though the quality of the data seemed as if, from what was proposed, would be really high, it felt like it would be hard to get many metrics included that would really tell us kind of what we would want to know (NP Community stakeholder 2).

Sometimes these more nimble and agile organisations can really facilitate what you’re trying to do, and these big grants, it would be great if they would like attach more comprehensively to an organisation that has the connections that we do to make stuff actually happen for us on the ground (NP community stakeholder 1).

Some food system stakeholders described previous engagement with less intensive research projects and were surprised by SALIENT’s level of detail and procedural requirements. Some expressed a preference for working with academics in a more advisory capacity, benefitting from their expertise, yet having control over their activities. Some organizations questioned the value of “gold-standard” methods for testing intervention effectiveness, particularly the use of randomization and control conditions. Community groups voiced concern that, if an intervention might be beneficial, access should not be restricted to create a control group. Some described randomizing individuals in need as both challenging and emotionally difficult and felt it resembled the “gamification” of food aid.

I felt for a brief moment, am I doing more harm than good? ’Cause you’ve come to us with the possibility you might get [the intervention]. We always say it’s random, it’s a 50/50 chance, but it’s hard for people to not get hopeful and then someone to leave feeling so disappointed. Like a middle-aged man on the verge of tears...” (participating community group interview).

Although SALIENT was funded in part by government departments, the majority of the grant was funded, and it was wholly administered by, the Economic and Social Research Council, which supported the independence of the research team. Both research team members and programme board participants described challenges in balancing the policy needs of government with the preservation of rigorous, independent scientific standards. Many programme board members were accustomed to, and preferred, a more directive relationship with researchers, often shaped by their experience of commissioning research. In SALIENT, some board members expressed a desire to influence the selection of interventions and the development of evaluation protocols. The research team, however, regarded this level of involvement as inappropriate, as they felt it risked compromising the independence of the research. These differing perspectives created tensions early in the project, with the programme board initially seeking decision-making authority and editorial control over outputs. These tensions were resolved through discussions but highlighted the importance of establishing clear roles and boundaries for each stakeholder group in collaborative research of this kind.

It has been a real eye opener about the subtle differences between policy requirements and academia requirements, you know, public health research requirements. Sometimes those differences are subtle and sometimes they’re not very subtle at all. We want to make sure that the work that we’re doing is robust, has academic value and academic merit, and I think the Programme Board, whilst they want all of those things, they also want to be able to kind of package it up and say, “We have influenced these groups to do this”, and I almost feel like they see that the research project is a slightly different thing (Consortium member 16).

#### Subtheme 3.2: The perceived risks associated with academic collaboration differ depending on food sector

All stakeholder groups highlighted the significant investment of time and resources required to engage fully in the co-design process and some described how these activities made it difficult for staff to focus on their regular duties. Some community sector organizations raised concerns about the short intervention periods and that interventions perceived by recipients as beneficial may not be continued after the study period. In addition, community organizations described requests to implement completely new projects as unrealistic, noting that more feasible collaboration should build upon their existing actions. Some food system stakeholders described the risk associated with the fact that not all proposed interventions were selected for evaluation and carried forward by the research team. Some felt clearer communication about project milestones and decision timelines would have helped manage their expectations and adjust their input levels.

I was a little bit miffed, I was like, “Come on, we’ve spent the best part of 3 or 4 months working with you to get it to this stage. I probably wouldn’t have done that if you’d said there was real risk that the board will turn us down (NP community stakeholder 3).

Participation in SALIENT required stakeholders to agree to publication of research findings regardless of what they were. Some commercial stakeholders saw this as risky, as it meant relinquishing control of communications and potentially limiting any commercial edge they may achieve by participation. Some also saw potential risk in being associated with “unsuccessful” trials.

They’re all questions we’d quite like to know the answer to, but we’d like to know the answer ourselves rather than doing it as part of broader research, so that somebody else knows or interprets the answers before we do… Others have done it very publicly and failed (NP commercial stakeholder 1).

#### Subtheme 3.3: The pace of academic research does not align with the pace of activities carried out by food system organizations

Many food system stakeholders described academic research as slow and not fitting with the pace of their organization. Academic processes such as ethical approvals, and in particular, contract requirements were described as causing substantial delays and were often a source of frustration. Whilst some commercial organizations described working on tight timelines with rapidly changing priorities, others discussed how many of their activities were planned many months in advance, making it difficult to co-design and implement an intervention in the limited funding period available. Others described entrepreneurial environments where decisions were made and acted upon rapidly and felt that this style of working was not replicated by the research team.

Just getting the funding signed off and all the ethics forms, that did take a long time. So, I guess maybe if those kind of processes could be streamlined. From our perspective, it’s a very entrepreneurial environment here and the co-CEOs and stuff, they’re always very keen for updates. So, it’s just like there was this real hunger and desire to find out and learn more. But we often had to just field off those kind of responses (participating commercial stakeholder 4).

We already have activity planning which is 18 months out, we’ve just seen Easter signed off, so again, we’re working quite far out, so those discussions don’t always align when you think they could or should, because we’re so far out in our timings. (NP commercial stakeholder 3).

The perceived slow pace of academic research was also noted by programme board members, who described sometimes wanting outputs from studies quickly, but this not aligning with the research team’s desire to gather robust evidence. The short time frame of SALIENT funding seemed to be more aligned with policy development than academic research.

I think one of the big tensions we have as a government department is that our timescales are really weird, so we often want things really fast and then won’t pick them up for 6 months. So, if a director says to us “you need to do some research on this, we will need information on this right now”, we’ll work really quickly and then things can often get forgotten very easily (PB member 4).

Participating stakeholders highlighted how long it took to finalize interventions details and research processes, which commercial stakeholders found particularly frustrating. However, some also acknowledged that they needed more time to develop their ideas and determine how the interventions would be implemented within their organizations. It was suggested that additional time could have helped identify greater shared benefits such as the inclusion of more meaningful outcomes for the food system partner.

## Discussion

### Summary of findings

This study explored how the SALIENT stakeholder engagement process works and what we can learn from it using a condensed theory of change (ToC) that described how collaborative, cross-sector food systems research was expected to function and the assumptions underpinning this process. Interpreting the findings through the ToC enabled us to identify which assumptions were realized, which were challenged and why, thereby providing an explanation of how and why stakeholder engagement worked for some organizations but not others, as well as identifying lessons for future collaborative food systems research.

Regarding the first research question on how and why the SALIENT process worked for some partners and not for others, stakeholder engagement was strongest where SALIENT objectives aligned with organizational priorities, where decision-makers with sufficient authority were engaged and where organizations perceived clear benefits from participation. However, engagement was constrained by competing organizational priorities, power imbalances between researchers and stakeholders and differences in the pace, purpose and expectations of research. These findings suggest that several assumptions underpinning the ToC, including stakeholder motivation, the development of trusting relationships and the co-design of interventions that were both feasible and scientifically valuable, were only partially realized because they were shaped by broader organizational and system-level constraints.

Regarding the second research question on what we can learn from this process, the evaluation identified several lessons for future collaborative food systems research. Long-term government leadership, stronger accountability and incentives for stakeholder engagement, sufficient time to build relationships and governance structures that protect research independence whilst supporting meaningful collaboration appear essential. The findings also suggest that co-design processes should explicitly balance stakeholder priorities with the need to generate evidence that addresses important uncertainties, whilst adopting evaluation approaches that are both scientifically rigorous and responsive to stakeholder needs. These findings also resonate with broader reflections from elsewhere, which emphasize that successful interdisciplinary food systems research depends on sustained relationship-building, effective collaboration across disciplinary and organizational boundaries, and research systems that provide sufficient time and flexibility for these processes to develop [24].

## Strength and limitations

This study included a broad range of perspectives, including researchers, government funding body representatives and stakeholders from community and commercial food sector organizations. This allowed us to explore the multiple factors influencing cross-sectorial collaboration within food systems research. Whilst we aimed to include as many stakeholders as possible, not all participating partners are represented in this study.

We used purposive sampling to ensure participation of different stakeholder types, sectors and levels of engagement, but several challenges affected recruitment. Some stakeholders were unresponsive or hesitant to participate, whilst others were already in ongoing discussions regarding future projects, and the team was reluctant to burden them with additional requests. Consequently, some perspectives may be underrepresented. Commercial participants who engaged were mainly from health and sustainability-focused teams and may represent only one organizational perspective. Insights from those working in other teams, such as sales or marketing, may have differed. At times, it was difficult to discern whether participants were speaking from personal experience or representing an organizational stance. In reality, their accounts likely reflected a mixture of both, shifting according to the issue at hand.

Contextual features of the UK food system, research structures and funding mechanisms may limit the transferability of our findings. Some interviews occurred more than a year after involvement in SALIENT activities, potentially affecting recall; however, our focus extended to broader perspectives on food system transformation and research. As interviews were conducted before the end of the SALIENT project, some later reflections may not have been captured.

Finally, the primary analysis was conducted by one researcher (S.S.). Although this is consistent with reflexive thematic analysis, the developing interpretation was discussed regularly with the wider research team to challenge assumptions and refine themes. We recognize the inherent challenges of critiquing and evaluating our own research. To reduce bias associated with this process, the lead author joined SALIENT after stakeholder engagement activities had commenced and was therefore able to provide greater analytical distance from many of the relationships and decisions being evaluated.

## Interpretations and implications

### Establishing a common goal and gaining commitment

Participants reported a wide range of actions relating to health and sustainability being taken by their organizations. However, this diversity of actions makes it difficult to achieve coherent progress towards sustained food system change. The inherent complexity of the food system creates multiple potential intervention points. However, research framed around the commercial determinants of health has shown that such complexity is often emphasized by commercial organizations, particularly those in health harming industries, as a justification for inaction or as a basis for resisting public health policies [25]. To move beyond fragmented efforts and towards more integrated approaches aimed at food system change, our findings highlight the importance of a long-term strategic direction that secures commitment across the food sector. Governments have an important role to play in providing this shared vision and commitment. Our findings align with previous calls to rethink and introduce more meaningful food system indicators of success, alongside enhanced accountability to address the drivers of poor diet and obesity [26].

Participants noted that discussions about interventions addressing sustainability tended to generate higher levels of engagement than those around health, particularly with commercial stakeholders. This was largely because organizations are already subject to stronger legal, reporting and policy-driven requirements on sustainability than on health outcomes. The UK Government’s recently published *10-Year Health Plan* [17] proposes mandatory reporting on healthy food sales, with ambitions to set future mandatory targets whilst allowing organizations flexibility in how they achieve these. Previous research has consistently shown that voluntary actions are largely ineffective at delivering meaningful changes in the food system [27, 28]. Our findings suggest that if such mandatory reporting requirements are implemented and future targets are adequately ambitious and enforced, they could emulate the apparent motivating effects of current sustainability targets. We also found that companies demonstrating the strongest motivation to participate in this independent research were often B-Corp affiliated, as engagement in academic research contributes to certification. In contrast, organizations that could not demonstrate tangible immediate benefits from participation were more reluctant to engage. These findings highlight the importance of measurable, externally set targets that have meaningful accountability processes for securing commercial commitment to both sustainability and health actions and related research. The Government’s Food Strategy for England (2025) emphasizes the need for strong research and development to transform the food system [29]. Our findings suggest that tangible incentives may be necessary to ensure participation in this research from across the food system. Such incentives could include tax relief for organizations that participate in high-quality independent food systems research, such as those provided for participation in science and technology research and development [30].

### The ability of co-design to address critical uncertainties

Our findings strongly align with previous work that has described the need to embrace the “messiness” of co-designed food systems research and to follow principles relating to relationships, power management and the valuing of diverse forms of knowledge [10]. However, recent critiques argue that collaborative research should move beyond treating all stakeholders as equivalent participants, recognizing instead that stakeholders differ substantially in their power, influence and ability to shape research agendas and outcomes [31]. Whilst a flexible and pragmatic approach to co-design can build stakeholder trust and engagement, it may also compromise the ability of subsequent evaluations to address critical uncertainties in existing knowledge. In SALIENT, researchers described being reluctant to pursue interventions that they believed, based on prior evidence, were unlikely to be effective, even though those were often considered the most feasible by large-scale organizations. This finding aligns with evidence which explored public attitudes to government health-related interventions and showed less intrusive interventions are generally more acceptable than more restrictive approaches to behaviour change [32]. In other cases, stakeholders had strong views on which interventions to implement and how to evaluate them, limiting researchers’ ability to address uncertainties in the literature; this aligns with research showing that commercial interests can shift agendas away from questions most relevant to public health [33]. Our findings illustrate how unequal power between stakeholder groups shaped both the interventions that were considered feasible and the evidence that could ultimately be generated, supporting calls for greater attention to power dynamics in collaborative research [31]. Nevertheless, what constitutes a “critical uncertainty” is itself shaped by stakeholders’ differing priorities, objectives and positions within the food system. Recognizing these differences, whilst remaining attentive to unequal power to define research agendas, is likely to be essential for future collaborative food systems research.

### Evaluations to meet stakeholder needs

Food system and policy stakeholders tended to value practice-oriented research that supported performance, whether market success or organizational efficiency, over the more time-intensive academic evaluations proposed by the research team. This priority often conflicted with researchers’ goals of producing robust, generalizable evidence. Previous evaluations of collaborative research have recommended adapting methodologies to support effective joint working and ensuring outcomes relevant to stakeholders are assessed at appropriate timepoints [12]. Conducting evaluability assessments with the input of all stakeholders early in a collaborative research project may be one way to help ensure the evidence generated is insightful and meaningful to all stakeholders [34]. Evaluability assessments may also make it possible to identify a wider range of outcomes that provide valuable insights to partners beyond those that may be of most interest to the research team. In addition, whilst some interventions may be amenable to randomized controlled trials, detailed process evaluations and modelling of long-term impacts, others may benefit from alternative, more rapid evaluative approaches which can be better aligned with stakeholder timelines and contexts [35, 36]. Whilst rapid research methods often receive the label of “quick and dirty”, they do not have to be, especially if robust routine data streams can be exploited [37]. Evaluability assessments may help to untangle these differences and may help to identify where research teams can provide unique expertise. Large-scale consortium projects, with their substantial resources and breadth, may be particularly well placed to support this diversity in knowledge generation, provided adequate time and flexibility are built into their design.

### Establishing effective relationships

Underpinning any effective collaborative project is the issue of establishing and maintaining strong relationships with all participating stakeholders. Numerous studies have reflected on the process of building effective working relationships [13, 38–41]. In our study, all stakeholder types acknowledged the large time investment necessary to develop effective working relationships. It is essential that research teams are strategic in developing, prioritizing and maintaining relationships [39]. Published guidance for conducting healthy food retail research highlights the necessity of having a clear understanding of potential collaborating organizations, including what motivates them and where there might be shared objectives [39]. Our research additionally clarifies that identifying and understanding the specific benefits of research involvement to stakeholders can help secure their participation. It may also help researchers focus their efforts on the potential collaborations most likely to bear fruit.

Another strategy to build strong relationships with food system stakeholders is to ensure that research teams include members who understand stakeholder priorities and language. In SALIENT, researchers recognized that many of their main contacts were within health and sustainability teams, and discussions often faltered because more commercially focused teams were not engaged early in the co-design process. Involving researchers with expertise in commercial priorities, such as business, supply chains and marketing, as well as in non-profit organizations, may help build stronger connections and foster more effective collaborations. These colleagues could act as brokers in negotiations between stakeholders and the wider research team [41].

In SALIENT, many relationships with participating food system organizations predated the project. Researchers should recognize that initial contact with new organizations may not immediately result in a “successful” partnership. Instead, a longer-term strategy is needed to sustain contact, build trust and deepen mutual understanding for future collaboration. This again highlights the need for sustained, longer-term funding to support this kind of collaborative work. Similar conclusions have been drawn elsewhere, where researchers identified relationship-building, interdisciplinary collaboration and systems change as processes that require investment beyond conventional research funding cycles [24].

Previous reflections from collaborative studies have stressed the importance of robust governance to safeguard research independence [41]. In SALIENT, delays in these processes were a source of considerable frustration and created significant challenges given the project’s short funding period. Ensuring adherence to ethical standards and establishing strong governance structures for data sharing and research independence are essential. It is therefore critical that universities and funding bodies ensure that adequate time, funding and resources are allocated to these processes to avoid unnecessary delays. Finally, establishing effective methods of communication is also essential for maintaining strong stakeholder relationships. Previous research has recommended developing communication plans for each partnership to ensure stakeholders receive regular updates [40]. In SALIENT, some researchers reflected that poor communication resulted in some stakeholders feeling let down, undermining the potential for future collaborations. Communication plans may assist with managing stakeholder expectations by ensuring study processes and timelines are thoroughly outlined from the outset.

### Recommendations for future collaborative food systems research

Drawing on the findings from this study, we propose several recommendations for researchers, funders, universities, policymakers and participating organizations seeking to undertake cross-sector food systems research (Table 3).

**Table 3:** Recommendations for future collaborative food systems research

Challenge identified in SALIENT	Recommendation	Key actors
Lack of shared priorities across food system actors	Develop long-term national food system strategies with measurable health and sustainability objectives to provide a shared direction for research and action	Government departments, policymakers
Limited incentives for commercial engagement in independent research	Establish clearer expectations and incentives for participation in high-quality independent food systems research, including consideration of reporting requirements, processes for recognition or financial incentives	Government, funders, industry bodies
Short funding cycles constrain relationship-building and collaboration	Provide longer-term funding models that recognize collaboration, partnership development and governance as essential research activities rather than administrative overheads	Research funders, universities
Governance and contracting processes delay collaboration	Allocate sufficient time and resources at project initiation for ethical review, data governance, legal agreements and stakeholder engagement processes	Universities, funders, research teams
Engagement was easier with health/sustainability teams than commercial functions	Broaden engagement beyond health, sustainability and corporate responsibility teams by involving individuals with expertise in commercial decision-making, supply chains, procurement and marketing	Researchers, partner organizations
Stakeholder priorities sometimes conflicted with scientific objectives	Establish shared expectations at the outset regarding research independence, evidence standards, intervention feasibility and stakeholder priorities	Researchers and partners
Different expectations of evaluation created tensions	Conduct early evaluability assessments to identify outcomes relevant to stakeholders and determine the most appropriate evaluation methods	Researchers, stakeholders
Communication challenges affected trust	Develop partnership-specific communication plans outlining timelines, responsibilities, decision-making processes and opportunities for feedback	Research teams and stakeholders

## Conclusions

This study addressed how and why stakeholder engagement functioned within a collaborative, co-designed food systems research programme, and identified lessons for future collaborative research. It highlights both the opportunities and challenges of pursuing collaborative, co-designed research to encourage healthy, sustainable diets. In relation to how stakeholder engagement functioned, participants felt that SALIENT generated valuable proof-of-principle evidence and strengthened understanding of the time, resources and relationships required for effective stakeholder collaboration. However, the project also exposed tensions between academic processes and the practical priorities of commercial, community and policy partners. Issues of power, ability to address critical uncertainties, timelines and governance created challenges to achieving ambitious interventions, whilst short project timeframes limited the depth of relationships that could be built. In relation to future collaborative food systems research, these findings underscore the importance of long-term strategic direction from government, stronger accountability mechanisms and tangible incentives to secure meaningful research engagement from organizations across the food system. Future large-scale collaborative projects need to balance flexibility with rigour, develop trust through sustained relationships and ensure that processes for governance, communication and evaluability are embedded from the outset. In doing so, they will be better positioned to generate robust, relevant and scalable evidence capable of driving meaningful change within the food system.

## Supplementary Information

Additional file 1.

Additional file 2.

## Data Availability

The datasets used and/or analysed during the current study are available from the corresponding author on reasonable request.
