# Supplementary material for: Conducting cross-sectoral collaborative research for food system transformation: learnings from a qualitative evaluation study
Source: Health Res Policy Syst. 2026 Oct 6;24:87. doi: 10.1186/s12961-026-01540-y (PMC13659941; doi:10.1186/s12961-026-01540-y)
Supplement: Supplementary file 1 [file 12961_2026_1540_MOESM2_ESM.docx]

**Topic Guide: Stakeholder interviews**

**Study Title:** SALIENT evaluation: Interviews with Stakeholders

**University of Cambridge sponsorship reference number: 24.360**

**Name of Researchers:** Dr Sarah Shaw, Prof Jean Adams, Prof Steve Cummins, Prof David Ogilvie, Prof Martin White

**Study overview**

The SALIENT consortium has been working with government, industry and non-profit sectors to develop, deliver and evaluate a range of co-designed interventions aiming to make the food system healthier and more environmentally sustainable.

In this interview, I would like to hear about your experience and perspective of being involved with SALIENT. This is your opportunity to provide feedback, not only to the research team but also the government and funders. Your participation today is voluntary, and you are free to not answer any questions or end the interview at any point.

Finally, I would like to record our conversation today. This recording will be transcribed and any information that may make you identifiable will be removed. We will keep your participation confidential and nothing you say today will be feedback to your employer. In write-ups and presentations about this research, we may use anonymised quotes.

**Verbal consent check**

Can I confirm you have read and understood the information about the study?

Have you completed the online consent form?

Do you have any questions before we proceed?

Are you happy for me to record this interview?

**Programme Board questions**

*About SALIENT*

- Tell me about your current role and how you’ve come to be involved with SALIENT
- What other experience do you have of/with academic research?
  - Do you have any experience of running real-world food trials?
- What do you consider to be the main aims/objectives of SALIENT?

*Interventions and expectations*

- What were your/ your department’s expectations when designing/commissioning the food trials funding call?
- How well do you think SALIENT has met these expectations?
- How has the experience of running real-work trials through SALIENT differed from your expectations before the project started?
- What are your thoughts on the range of interventions that have been delivered through SALIENT? *[researcher to refer to list of interventions if needed]*
  - In general terms, how might these interventions change behaviour?
  - How innovative do you think the interventions are?
  - How do they align with your previous expectations?
  - What are your thoughts on the range of organisations/businesses that have engaged with SALIENT? [provide list if necessary]
- How well has SALIENT aligned with the strategic direction of your department?
- How often does your department use scientific evidence in your work?
- What do you consider to be the possible policy implications of SALIENT’s research findings?
- What lessons do you think should be learnt from SALIENT?
  - For the food industry?
  - For other food system stakeholders (charity and community sectors)?
  - For government?
  - For researchers?

*Food Systems Change*

- In your opinion, what initiatives are needed in the future to achieve a healthier, more equitable and more sustainable food system?
  - How should we decide on priority areas?
- What do you consider to be the biggest barriers to food systems change?
- What role do you feel the food industry could, or should, have in achieving a healthier, more equitable and more sustainable food system?
- Do you have any other feedback for the research team?

**Food system stakeholder questions (For those who took part)**

*About SALIENT*

- Tell me about your current role and how you’ve come to be involved with SALIENT
- What experience did your organisation have in participating in research prior to SALIENT?
- What do you consider to be the main aims/objectives of SALIENT?
- What were the main motivations for your organisation working with SALIENT?
  - Did you have any previous contact/interactions with the research team?
  - Does your organisation have an Environment, Social and Governance (ESG) or Carbon strategy?
- What are your perspectives on participating in government funded research?
  - What are the benefits for your organisation?
  - What are the drawbacks for your organisation?
  - How useful do you consider this type of research to be to your organisation?
  - How often do you use research findings in your work activities?

*Co-design and intervention*

- Can you briefly describe the intervention that was introduced in your organisation?
  - How were the final intervention components decided upon?
  - Can you tell me a bit about the process of negotiating the intervention with the research team?
  - How did the co-design process work?
  - Who from your company was involved?
  - What difficulties were experienced during the co-design process?
  - What went well during the co-design process?
  - How closely did the goals of your business align with those of the research team?
- What impact has this intervention had?
  - What do you consider to be the potential impacts on health and sustainability?
  - What are the business impacts?
  - Expected/unexpected impacts
- How likely are you to continue the intervention in your organisation?
- If your organisation were to take part in a programme like SALIENT in the future, what do you think could be improved?
  - What additional support might be needed?
- How likely would you be to engage in this sort of co-design initiative in the future?

*Food system changes*

- In your opinion, what initiatives are needed in the future to achieve a healthier, more equitable and more sustainable food system?
  - How should we decide on priority areas?
- What role do you feel organisations like yours have in achieving a healthier, more equitable and more sustainable food system?
- What do you consider to be the biggest barriers to food systems change?
- Do you have any other feedback for the research team or government?

**Food system stakeholder questions (For those who withdrew)**

*About SALIENT*

- Tell me about your current role
- Tell me about the interactions you have had with the SALIENT research team
  - How did you first become aware of SALIENT?
  - Have you had any previous interactions with the research team
- What do you consider to be the main aims/objectives of SALIENT?
- What experience does your organisation have in participating in research studies?
- What were the main motivations for your organisation considering taking part in SALIENT?
  - Does your organisation have an Environment, Social and Governance (ESG) or Carbon strategy?
- What are your perspectives on participating in government funded research?
  - What are the benefits for your organisation?
  - What are the drawbacks for your organisation?
  - How useful do you consider this type of research to be to your organisation?
  - How often do you use research findings in your work activities?

*Co-design and intervention*

- How far did your company progress in terms of considering to take part/co-designing an intervention with the research team?
  - *[Researcher to know where partner dropped out]*
  - How did the co-design process work?
  - Who from your company was involved?
- What were your organisation’s main reasons for withdrawing from SALIENT?
  - How was this decision made?
  - Did anything go badly during your discussions with the research team?
  - Did anything go well during our discussions with the research team?
- How likely would you be to engage in this sort of co-design initiative in the future?
  - What could be done differently to make you more likely to take part in this sort of initiative in the future?

*Food Systems Change*

- In your opinion, what initiatives are needed in the future to achieve a healthier, more equitable and more sustainable food system?
  - How should we decide on priority areas?
- What role do you feel organisations like yours have in achieving a healthier, more equitable and more sustainable food system?
- What do you consider to be the biggest barriers to food systems change?
- Do you have any other feedback for the research team or government?

**Research team questions**

*About SALIENT*

- Tell me about your current role in the SALIENT programme
  - Which food system partners did you work with?
  - What elements of the SALIENT process have you been involved with? [co-design, implementation, evaluation, other]
- What do you consider to be the main aims/objectives of SALIENT?

*Co-design, interventions and evaluations*

- How did you first make contact with the food systems partners about SALIENT?
  - How did the initial discussions about the project go?
  - What worked well at this stage of the process?
  - What were the main challenges at this stage of the process?
  - What motivations/hesitations did food systems partners have in relation to participation at this stage?
  - What do you feel were the main reasons partners decided to withdraw from SALIENT? [ask if applicable]
- How were the interventions decided upon? [ask about all intervention that the participant was involved with]
  - Was it co-designed/ a retrospective evaluation?
- How did the co-design/ implementation/ evaluation process go? [Discuss all that are appropriate]
  - Can you briefly describe the co-design/ implementation/ evaluation process?
  - What worked well?
  - What were the challenges?
  - What efforts were made to overcome these challenges?
  - How aligned were the partners and the research team?
  - How invested were the partners in the SALIENT process?
- What do you feel could be improved if the SALIENT process was repeated in the future?

Food Systems Change

- What do you consider to be the biggest barriers to food systems change?
- What do you consider to be the biggest unmet challenges that need to be addressed?
- What role do you feel food system stakeholders could, or should, have in achieving a healthier, more equitable and more sustainable food system?

Do you have any feedback for the government, funders or food system stakeholders?

**Post-interview checklist for interviewer:**

**☐** mp4 recording file converted to mp3 audio file

**☐** mp3 **a**udio recording uploaded to SRD and checked that the voices are clear, etc.

**☐** Transcript downloaded from Teams and uploaded to SRD

☐ Recording and transcript deleted from OneDrive/Teams

**In person interviews or when Dictaphone has been used**

**☐** Audio recording uploaded to SRD and checked that the voices are clear, etc.

**☐** Transcript request form completed and sent

**☐** Audio recording deleted from Dictaphone.
